# Temperature effects on gene expression and morphological development of European eel, *Anguilla anguilla* larvae

**DOI:** 10.1371/journal.pone.0182726

**Published:** 2017-08-14

**Authors:** Sebastian N. Politis, David Mazurais, Arianna Servili, Jose-Luis Zambonino-Infante, Joanna J. Miest, Sune R. Sørensen, Jonna Tomkiewicz, Ian A. E. Butts

**Affiliations:** 1 National Institute of Aquatic Resources, Technical University of Denmark, DTU, Lyngby, Denmark; 2 Ifremer, Marine Environmental Science Laboratory UMR 6539, Plouzané, France; 3 Helmholtz Centre for Ocean Research, GEOMAR, Kiel, Germany; Institute of Marine Research, NORWAY

## Abstract

Temperature is important for optimization of rearing conditions in aquaculture, especially during the critical early life history stages of fish. Here, we experimentally investigated the impact of temperature (16, 18, 20, 22 and 24°C) on thermally induced phenotypic variability, from larval hatch to first-feeding, and the linked expression of targeted genes [heat shock proteins (*hsp*), growth hormone (*gh*) and insulin-like growth factors (*igf*)] associated to larval performance of European eel, *Anguilla anguilla*. Temperature effects on larval morphology and gene expression were investigated throughout early larval development (in real time from 0 to 18 days post hatch) and at specific developmental stages (hatch, jaw/teeth formation, and first-feeding). Results showed that hatch success, yolk utilization efficiency, survival, deformities, yolk utilization, and growth rates were all significantly affected by temperature. In real time, increasing temperature from 16 to 22°C accelerated larval development, while larval gene expression patterns (*hsp70*, *hsp90*, *gh* and *igf-1*) were delayed at cold temperatures (16°C) or accelerated at warm temperatures (20–22°C). All targeted genes (*hsp70*, *hsp90*, *gh*, *igf-1*, *igf-2a*, *igf-2b*) were differentially expressed during larval development. Moreover, expression of *gh* was highest at 16°C during the jaw/teeth formation, and the first-feeding developmental stages, while expression of *hsp90* was highest at 22°C, suggesting thermal stress. Furthermore, 24°C was shown to be deleterious (resulting in 100% mortality), while 16°C and 22°C (~50 and 90% deformities respectively) represent the lower and upper thermal tolerance limits. In conclusion, the high survival, lowest incidence of deformities at hatch, high yolk utilization efficiency, high *gh* and low *hsp* expression, suggest 18°C as the optimal temperature for offspring of European eel. Furthermore, our results suggest that the still enigmatic early life history stages of European eel may inhabit the deeper layer of the Sargasso Sea and indicate vulnerability of this critically endangered species to increasing ocean temperature.

## Introduction

European eel (*Anguilla anguilla*) aquaculture is capture-based, relying on wild-caught juvenile glass eels entering coastal waters, which are farmed until marketable sizes. However, historically low stock levels and failing recruitment [1] render this practice unsustainable and therefore establishment of breeding technology and larvi-culture is required for future aquaculture of this critically endangered species [2]. By, modifying the hormonal treatments from Japanese eel (*Anguilla japonica*) protocols, recent advances in assisted reproduction of European eels have led to a stable production of eggs and larvae, forming the basis of development of larval culture technology and first-feeding protocols [3]. In order to establish another promising step towards sustainable aquaculture of this species, it is necessary to identify optimal rearing conditions for early life history (ELH) stages.

The choice of rearing conditions used in aquaculture for a particular species is commonly based on either ambient conditions in their natural environment or experimental findings. In the case of European eel, the natural environmental regimes of embryos and the earliest larval stages (pre-leptocephalus) remain unclear. The spawning area of this species has been delimited to the western Atlantic Ocean (Sargasso Sea) and validated by the occurrence of the so far earliest larval stages found in nature [4–6]. Thus, eel larvae are believed to initiate their migration journey from the Sargasso Sea, a water mass characterized by a rather constant salinity of 36.5 ppt, a seasonal thermocline (~18°C) at a depth of 200–300 m and a warm water (20–28°C) upper layer zone [6, 7]. Thereafter, towards the European continent, glass eels inhabit temperate regions of a wide range of latitudes and longitudes with temperatures spanning between 2 and 28°C [8, 9].

In the past decades, there has been an increasing interest in eel research, especially concerning assisted reproduction and subsequent ELH rearing conditions in aquaculture. Thus, breeding protocols using assisted reproduction were developed for the Japanese eel in the 1970s [10], leading to the first glass eel production in recent years [11]. Since then, several studies have focused on optimizing rearing conditions of Japanese eel larvae, including the identification of salinity regimes or thermal tolerance ranges and limits during ELH stages [12–15]. In more detail, it was shown that a 50% reduction of the original seawater salinity resulted in increased offspring growth and survival performance [12]. Additionally, early ontogeny was found to be also influenced by temperature, showing suboptimal performance towards colder and warmer temperature limits (16–31°C), with optimum thermal conditions (~25°C) similar to those found in the recently identified natural spawning area of the Japanese eel [15, 16]. Moreover, induced maturation, *in vitro* fertilization, and early development of the American eel (*Anguilla rostrata*) have been reported, with larvae surviving up to 6 days post-hatch (dph) when reared at 20°C [17]. Furthermore, eel hybrids have gained attention as an alternative for the difficult assisted reproduction practices in aquaculture. Hybrids between *A*. *anguilla* and *A*. *rostrata* occur naturally since they share spawning grounds [18], though hybrids between male *A*. *anguilla* and female *A*. *japonica* have also been experimentally produced and their endogenously feeding larvae developed for 9 dph at 21–22°C [19]. Captive breeding has also been attempted with the long-finned (*A*. *dieffenbachii*) and short-finned (*A*. *australis*) eels. Though, successful hatching has only been reported for *A*. *australis* or the *A*. *australis* × *A*. *dieffenbachii* hybrid, under a thermal regime of 18.2–22.7°C [20]. Moreover, production of hybrid larvae from male *A*. *anguilla* and female *A*. *australis* and their survival for up to 5 dph was reported when fertilized and reared at 20–21°C [21]. Nevertheless, the thermal tolerance ranges of all the above species remain to be further investigated.

Identifying the optimal thermal conditions for rearing of eel ELH stages and establishing hatchery practice will benefit the future aquaculture industry, commonly targeting high production efficiency where high survival and growth potential are fundamental to a cost-effective production. Relatively small changes in rates of growth and mortality during embryogenesis and/or larval ontogeny can significantly influence reproductive success [22] and thus production efficiency in aquaculture. Temperature controls fundamental biochemical processes, thereby influencing developmental rates and survival of marine fish larvae [23]. Especially during early development, teleost offspring can be stenothermal and be profoundly affected by even minor temperature changes [24, 25]. Moreover, their ELH stages are influenced physiologically by extrinsic factors such as temperature and their interaction with intrinsic properties endowed to them by their parents [24, 26].

Today molecular methods and tools are increasingly accessible and economically affordable. Whole species genomes are publically available, including the European eel genome, which was recently sequenced and assembled [27–28]. This offers new perspectives for eel research, such as using RNA sequencing to identify actors involved in different biological processes, cellular components and molecular functions that are of importance in this species [29]. As such, in order to molecularly understand phenotypic sensitivity of ELH stages to extrinsic environmental factors (such as temperature), it is now possible to follow expression of targeted genes controlling the development of this fish species [30, 31]. For instance, heat shock proteins (HSP) play a fundamental role in the regulation of normal protein synthesis within the cell and are critical to the folding and assembly of other cellular proteins [30]. As such, it is hypothesized that an up-regulated expression of fish larval *hsp*’s, would be associated with vulnerability to thermal injury, as the *hsp* response is a cellular mechanism activated to prevent cell damage caused by thermal stress [32]. Moreover, fish communicate with their physiologic environment, by using the somatotropic axis, a mechanism combining growth hormones (GH) and the closely connected and regulated by GH, insulin-like growth factors (IGF), that are involved in most physiological processes including metabolism and growth [31]. Here, it is hypothesized that the activation of the GH/IGF system triggering cell proliferation and DNA synthesis, could be stimulated by temperature and the up-regulated expression response would be associated among others with improved growth, metabolism and development [31, 33].

In this context, we experimentally investigated the impact of temperature on European eel larvae from hatching to first-feeding, through an integrative morphological and molecular approach. Thus, the objectives of this study were i) to identify the thermal tolerance range and limits for development and growth of European eel larvae, and ii) to elucidate thermally induced phenotypical changes and the interlinked gene expression of genes (*hsp’s*, *gh* and *igf’s*) involved in molecular mechanisms commonly associated to fish early life development.

## Materials and methods

### Ethics statement

All fish were handled in accordance with the European Union regulations concerning the protection of experimental animals (Dir 86/609/EEC). Eel experimental protocols were approved by the Animal Experiments Inspectorate (AEI), Danish Ministry of Food, Agriculture and Fisheries (permit number: 2012-15-2934-00458). Briefly, adult eels were anesthetized using ethyl p-aminobenzoate (benzocaine) before tagging and handling. Endogenously feeding larvae of European eel were anesthetized prior to handling and euthanized prior to sampling by using tricaine methanesulfonate (MS-222). All efforts were made to minimize animal handling and stress.

### Broodstock management and gamete production

Female silver eels were obtained from a freshwater lake, Vandet, Jutland, Denmark. Male eels were obtained from a Danish commercial eel farm (Stensgård Eel Farm A/S). Experimental maturations were conducted at a DTU Aqua research facility at Lyksvad Fishfarm, Vamdrup, Denmark, where eels were housed in 300 L tanks equipped with a recirculation system [34]. Eels were maintained under low intensity light (~20 lux), 12 h day/12 h night photoperiod, salinity of ~36 ppt, and temperature of 20°C. Acclimatization took place over 10 days. As eels naturally undergo a fasting period from the onset of the pre-pubertal silvering stage, they were not fed during treatment. Prior to experimentation, eels were anaesthetized (ethyl p-aminobenzoate, 20 mg L^-1^; Sigma-Aldrich, Missouri, USA) and tagged with a passive integrated transponder. Females used for experiments (n = 4) had a mean (± SEM) standard length and body weight of 65 ± 4 cm and 486 ± 90 g, respectively. To induce vitellogenesis females received weekly injections of salmon pituitary extract (Argent Chemical Laboratories, Washington, USA) at 18.75 mg kg^-1^ body weight [11, 34]. To stimulate follicular maturation and induce ovulation, females received 17α,20ß-dihydroxy-4-pregnen-3-one (Sigma-Aldrich, Missouri, USA) at 2 mg kg^-1^ body weight [35] and were strip-spawned within the subsequent 12–14 h. Male eels (n = 11) had a mean (± SEM) standard length and body weight of 40 ± 3 cm and 135 ± 25 g, respectively. Males received weekly injections of human chorionic gonadotropin (Sigma-Aldrich, Missouri, USA) at 150 IU per fish [34]. Prior to fertilization, an additional injection was given and milt was collected by strip-spawning ~12 h after administration of hormone. Milt samples were pipetted into a P1 immobilizing medium [36] and only males with sperm motility of category IV (75–90%) were used for fertilization within 4 h of collection [37]. Only floating viable eggs/embryos were further used for experimentation.

### Experimental conditions

The experiment was repeated 4 times, within the same spawning season (2015), each time using a different parental cross. Eggs from each female were “crossed” with a sperm pool of several males to experimentally create 4 parental crosses. Eggs from each female were stripped into dry 36 × 30 × 7 cm plastic containers and gametes were swirled together while 0.2 μm filtered UV sterilized seawater was added for a gamete contact time of 5 min [37]. Seawater was obtained from the North Sea (~32.5 ppt) and temperature was adjusted to 20°C (± 0.1°C) while salinity was adjusted to 36 (± 0.1) ppt using Red Sea Salt (Red Sea Europe, Verneuil-sur-Avre, France) as previously defined [37, 38]. Egg density was determined by counting 3 × 0.1 mL subsamples of the floating layer. Within 30 min post fertilization, ~500 floating viable eggs/embryos per 100 mL, with a mean size (± SD) of 1.5 ± 0.1 mm (measured from images taken at 2 hpf), were distributed in replicated 600 mL flasks [182.5 cm^2^ sterile tissue culture flasks with plug seal caps (VWR^®^)] dedicated to larval sampling for morphology (3 replicates) and molecular analysis (2 replicates). Additionally, ~500 floating viable eggs/embryos per 100 mL were distributed in replicated (3×) 200 mL flasks (75 cm^2^ non-pyrogenic and non-cytotoxic flasks with plug seal caps, Sarstedt, Inc.) dedicated to sampling for hatch success and deformities at hatch. Seawater from the North Sea (0.2 μm filtered and UV sterilized), was adjusted to 36 (± 0.1) ppt (as above) and supplemented with rifampicin and ampicillin (each 50 mg L^-1^, Sigma-Aldrich, Missouri, USA) to increase survival as previously defined [39]. Embryos and larvae, from each parental cross, were reared in thermal controlling incubators (MIR-154 Incubator, Panasonic Europe B.V.) at five temperatures (16, 18, 20, 22 and 24°C ± 0.1°C). All experimental units were acclimatized to the treatment temperature within 1 h and salinity was kept at 36 (± 1) ppt. Temperature and salinity conditions of treatments were chosen to closely resemble the environmental conditions encountered at different depths of the assumed spawning areas in the Sargasso Sea [6]. Rearing of embryos and larvae took place in darkness while handling and sampling under low intensity (< 2.2 μmol m^-2^ s^-1^) light conditions as previously defined [40].

### Data collection

Larval development and gene expression were followed from hatch until the corresponding first-feeding stage in each temperature treatment. The first-feeding stage, as previously defined [3], was set as the time point when eye pigmentation, mouth and jaw formation was completed. Endogenously feeding larvae of European eel were anesthetized using tricaine methanesulfonate (MS-222) prior to digital imaging and euthanized post-sampling by using an MS-222 overdose. All images were taken using a digital camera (Digital Sight DS-Fi1, Nikon Corporation, Japan) attached to an objective microscope (Eclipse 55i, Nikon Corporation, Japan). NIS-Elements D analysis software (Version 3.2) was used to analyze the images of eggs, embryos, and larvae (Nikon Corporation, Japan).

#### Hatch success and deformities

Once hatching was completed, embryos and larvae within each experimental unit, dedicated to analysis of hatch success and deformities (× 3 replicates × 4 parental crosses) from each temperature treatment were digitally imaged for later classification. Embryos that were oversized, dark, discolored or exhibited abnormities in the cytoplasm were considered dead. Hatching success was then expressed as the total number of larvae divided by the total number of eggs. Larvae with abnormal and/or malformed head, body, yolk-sac or tail regions were classified as deformed.

#### Biometry

For analysis of larval morphology, ~15 larvae (× 3 replicates × 4 parental crosses) from each temperature were randomly sampled at hatch and every second day post-hatch. Larvae were digitally imaged for later analyses, where total length (from the tip of the snout to the posterior end of the caudal fin) and total yolk-sac area were measured from each larva. Larval growth and yolk utilization (YU) were measured from the change in length and yolk area from hatching until first-feeding. Yolk utilization efficiency (YUE) was measured by dividing the increase in length from hatching until first-feeding by the corresponding decrease in yolk area.

#### Molecular analyses

For molecular analysis, ~30 larvae (× 2 replicates × 4 parental crosses) from each temperature (16, 18, 20 and 22°C) were randomly sampled at hatch and every second day post-hatch until the first-feeding stage. Those larvae were not observed under the microscope (to avoid any influence on gene expression) but were immediately euthanized using MS-222, rinsed with deionized water, preserved in a RNA Stabilization Reagent and kept at -20°C following the procedure suggested by the supplier (Qiagen, Hilden, Germany).

For total RNA extraction, the larval pool (~30 larvae) of each replicate was homogenized in 800 μl Tri-Reagent (Sigma-Aldrich, Missouri, USA). After obtaining the aqueous phase by incubation in 160 μl chloroform, RNA was extracted using the InviTrap^®^ Spin tissue RNA MiniKit (STRATEC Biomedical AG, Berlin-Buch, Germany) following the manufacturer’s instructions. RNA concentration (764 ± 60 ng μl^-1^) and purity (260/280 = 2.12 ± 0.16, 230/260 = 2.16 ± 0.16) were determined by spectrophotometry using Nanodrop ND-1000 (Peqlab, Germany). From the resulting total RNA, 680 ng were transcribed using the Quanta qScript cDNA Synthesis Kit (Promega, Germany) according to the manufacturer’s instructions, including an additional gDNA wipe out step prior to transcription [Quanta PerfeCta DNase I Kit (Promega, Germany)].

The *ef1a*, *18s*, *40s* genes were chosen as housekeeping genes since qBase+ software revealed that these mRNA levels were stable throughout analyzed samples (M < 0.4); M gives the gene stability and M < 0.5 is typical for stably expressed reference genes [41]. The expression levels of target (*gh*, *igf-1*, *igf-2a*, *igf-2b*, *hsp70*, *hsp90*) and reference (*ef1a*, *18s*, *40s*) genes were determined by quantitative real-time PCR (qRT-PCR), using specific primers. Primers were designed using primer 3 software v 0.4.0 (http://frodo.wi.mit.edu/primer3/) based on predicted or cloned cDNA sequences available on Genbank databases (Table 1). Predicted cDNA sequences for *gh*, *igf-2a*, *igf-2b* and *18s* genes were deduced from genomic DNA sequences originated from the European eel genome [27]. All primers were designed for an amplification size ranging from 75 to 200 nucleotides.

**Table 1:** Sequences of European eel (*Anguilla anguilla*) primers used for amplification of genes by qRT-PCR. Primers were designed from predicted or cloned cDNA sequences available in Genbank nucleotide and WGS databases. The table lists accession number and corresponding database of target gene sequences.

Full name	Abbreviation	Databases	Accession Numbers	Primer sequence (5’_3’) (F: Forward; R: Reverse)
Heat Shock Protein 70	*hsp70*	GenBank	AZBK01685255	F: TCAACCCAGATGAAGCAGTG
				R: GCAGCAGATCCTGAACATTG
Heat Shock Protein 90	*hsp90*	GenBank	AZBK01838994	F: ACCATTGCCAAGTCAGGAAC
				R: ACTGCTCATCGTCATTGTGC
Growth Hormone	*gh*	GenBank WGS	AZBK01601863	F: TGAACAAGGGCATCAATGAA
				R: CGGAGCTTTCTCACATCCTC
Insulin-like Growth Factor-1	*igf-1*	GenBank Nucleotide	EU018410.1	F: TTCCTCTTAGCTGGGCTTTG
				R: AGCACCAGAGAGAGGGTGTG
Insulin-like Growth Factor-2-1	*igf-2a*	GenBank WGS	AZBK01717674	F: ACAACGGATATGGAGGACCA
				R: GGAAGTGGGCATCTTTCTGA
Insulin-like Growth Factor-2-2	*igf-2b*	GenBank WGS	AZBK01622663	F: AAAGCTTTGGGACAGCTTCA
				R: CGCAGCTGTGTACGTGAAAT
Elongation Factor 1-alpha	*ef1a*	GenBank Nucleotide	EU407824.1	F: CTGAAGCCTGGTATGGTGGT
				R: CATGGTGCATTTCCACAGAC
Ribosomal 18S RNA	*18s*	GenBank WGS	AZBK01681648	F: AGAGCAGGGGAACTGACTGA
				R: ACCTGGCTGTATTTGCCATC
Ribosomal 40S RNA	*40s*	GenBank TSA	GBXM01005349.1	F: TGACCGATGATGAGGTTGAG
				R: GTTTGTTGTCCAGACCGTTG

Expression of genes in each larval sample (2 biological replicates) were analysed in technical triplicates of each primer using the qPCR Biomark^TM^ HD system (Fluidigm) based on 96.96 dynamic arrays (GE chips) as previously described [42]. In brief, a pre-amplification step was performed with a 500 nM primer pool of all primers in TaqMan-PreAmp Master Mix (Applied Biosystems) and 1.3 μl cDNA per sample at 10 min at 95°C; 14 cycles: 15 s at 95°C and 4 min at 60°C. Obtained PCR products were diluted 1:10 with low EDTA-TE buffer. The pre-amplified product was loaded onto the chip with SSofast-EvaGreen Supermix low Rox (Bio Rad) and DNA-Binding Dye Sample Loading Reagent (Fluidigm). Primers were loaded onto the chip at a concentration of 50 μM. The chip was run according to the Fluidigm 96.96 PCR protocol with a Tm of 60°C. The relative quantity of target gene transcripts was normalized and measured using the ΔΔ Ct method [43]. Coefficient of variation (CV) of triplicates was calculated and checked to be < 0.04 [41]. If CV was found to be > 0.04, triplicates were checked for outliers and if possible duplicate measurements were used. If the use of duplicates was not possible (CV > 0.04) the whole data point was omitted from the analysis. In this automated analysis, malfunctions on the chip and/or unsuccessful RNA extractions can result in expression failures, as was the case in the Day 14 sample of 18°C in this study. Since qBase+ software revealed that *ef1a*, *18s* and *40s* mRNA levels were stable throughout the analyzed samples (M < 0.4), those genes were chosen as housekeeping genes.

### Statistical analyses

All data were analyzed using SAS statistical software (version 9.1; SAS Institute Inc., Cary, North Carolina). Residuals were tested for normality using the Shapiro–Wilk test and homogeneity of variances was tested using a plot of residuals versus fit values (PROC GLOT, SAS Institute 2003). Data were log_10_ or arcsine square-root-transformed when data deviated from normality and/or homoscedasticity [44].

#### Larval hatch success, survival, deformities, growth rate, yolk utilization, and yolk utilization efficiency

The effect of temperature on larval hatch success, survival, deformities, growth rate, yolk utilization, and yolk utilization efficiency was determined using a series of one-way ANOVA models, where parental cross was considered a random factor (SAS PROC MIXED; SAS Institute 2003). Tukey’s post-hoc analyses were used to compare least-squares means between treatments.

#### Larval morphology and molecular analyses

Statistical models were used to investigate temperature effects on larval morphology and gene expression throughout early larval development (Ages 0 to 18 dph) and at specific developmental stages (Stages 1–3). Across the different temperature treatments, Stage 1 represents the day of hatch, Stage 2 represents the timing of jaw/teeth formation and Stage 3 represents the first-feeding stages. Together this allowed us to decipher changes in temperature at standardized and real-time developmental intervals.

To examine the effect of temperature on larval morphology and gene expression throughout early development, we used two statistical approaches. In the first approach, we analyzed the data using a series of repeated measures mixed-model ANOVAs (PROC MIXED; SAS Institute 2003). Models contained temperature (16, 18, 20, 22 and 24°C) and age (0 to 18 dph) or stage (1, 2 and 3) main effects as well as the temperature × age (or stage) interaction. Akaike’s (AIC) and Bayesian (BIC) information criteria were used to assess which covariance structure (compound symmetry, autoregressive order, or unstructured) was most appropriate [45]. Temperature and age (or stage) were considered fixed, whereas parental cross was considered random. Tukey’s post-hoc analyses were used to compare least-squares means between treatments. If a significant temperature × age (or stage) interaction was detected, the model was decomposed into a series of reduced ANOVA models to determine the effect of temperature for each age (or stage) and of age (or stage) for each temperature. This was the case for length, yolk area, *gh*, *igf-1*, *hsp70* and *hsp90*. The reduced models involved only preplanned comparisons and did not include repeated use of the same data, so a-level corrections for a posteriori comparisons were not necessary.

In the second approach, we examined variation in larval morphology and gene expression, throughout development at each temperature, by fitting either linear, quadratic, cubic or exponential equations to the data (PROC REG; SAS Institute 2003). This allowed us to create predictive models to explore the shape of developmental variation for each temperature. Linear, quadratic, cubic and exponential equations were chosen a-priori to fit the data based on the available literature [46, 47]. Final equation selection (linear, quadratic, cubic or exponential) was based on an F-statistic: d.f._j_ × (R^2^_j_−R^2^_i_) / (1 –R^2^_j_), where: R^2^_i_ = the R^2^ for the i-th order, R^2^_j_ = the R^2^ for the next higher order, d.f._j_ = the degrees of freedom for the higher-order equation with j degrees of freedom in the numerator and d.f._j_ = n − j −1 degrees of freedom in the denominator [47]. Graphs and regressions were prepared in SigmaPlot (Version 13.0).

## Results

### Larval development, hatch success, survival and deformities

**Fig 1:**
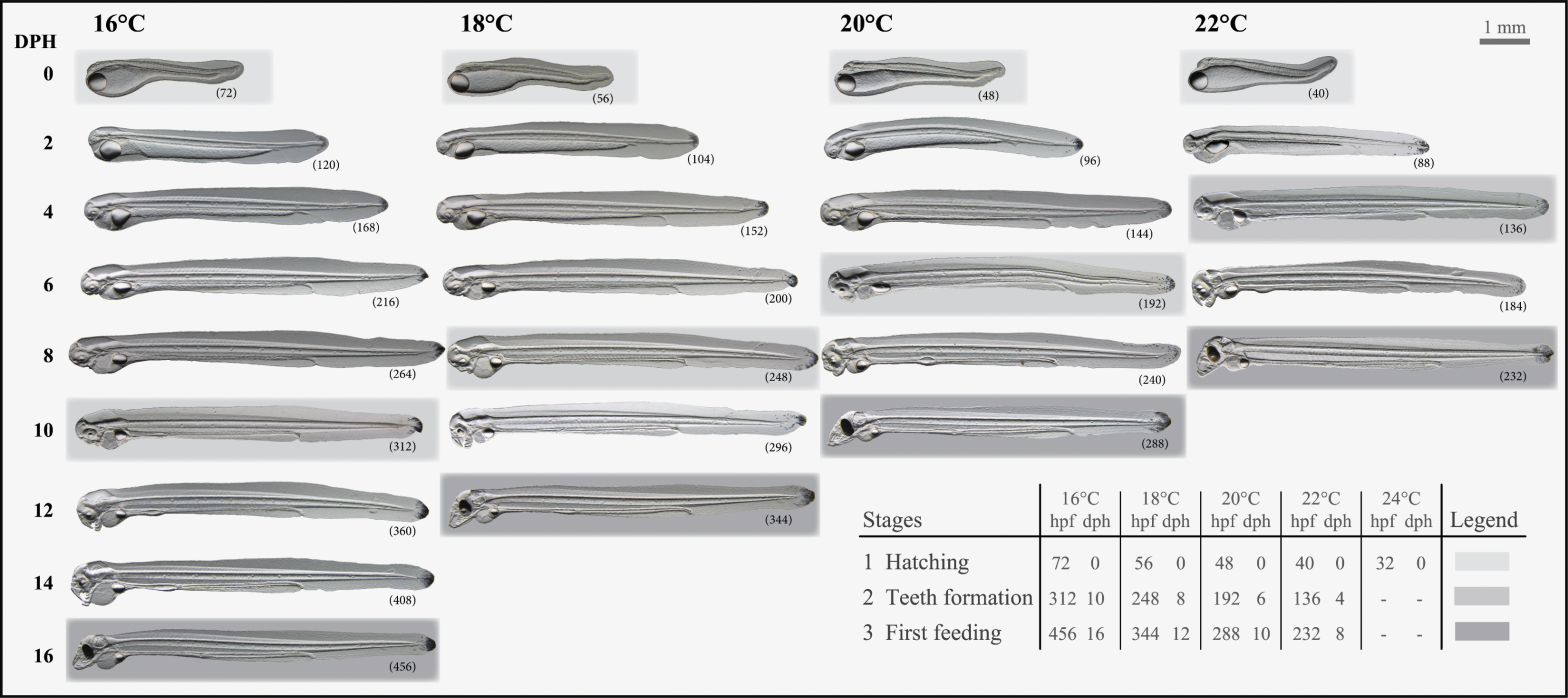
Timing of morphological features during development of larval European eel (*Anguilla anguilla*). Larval age presented in hours post-fertilization (hpf) and days post-hatch (dph) for five rearing temperature regimes. Across the different temperature treatments, Stage 1 represents the day of hatch, Stage 2 represents the timing of jaw/teeth formation, while Stage 3 represents the first-feeding stages.

**Fig 2:**
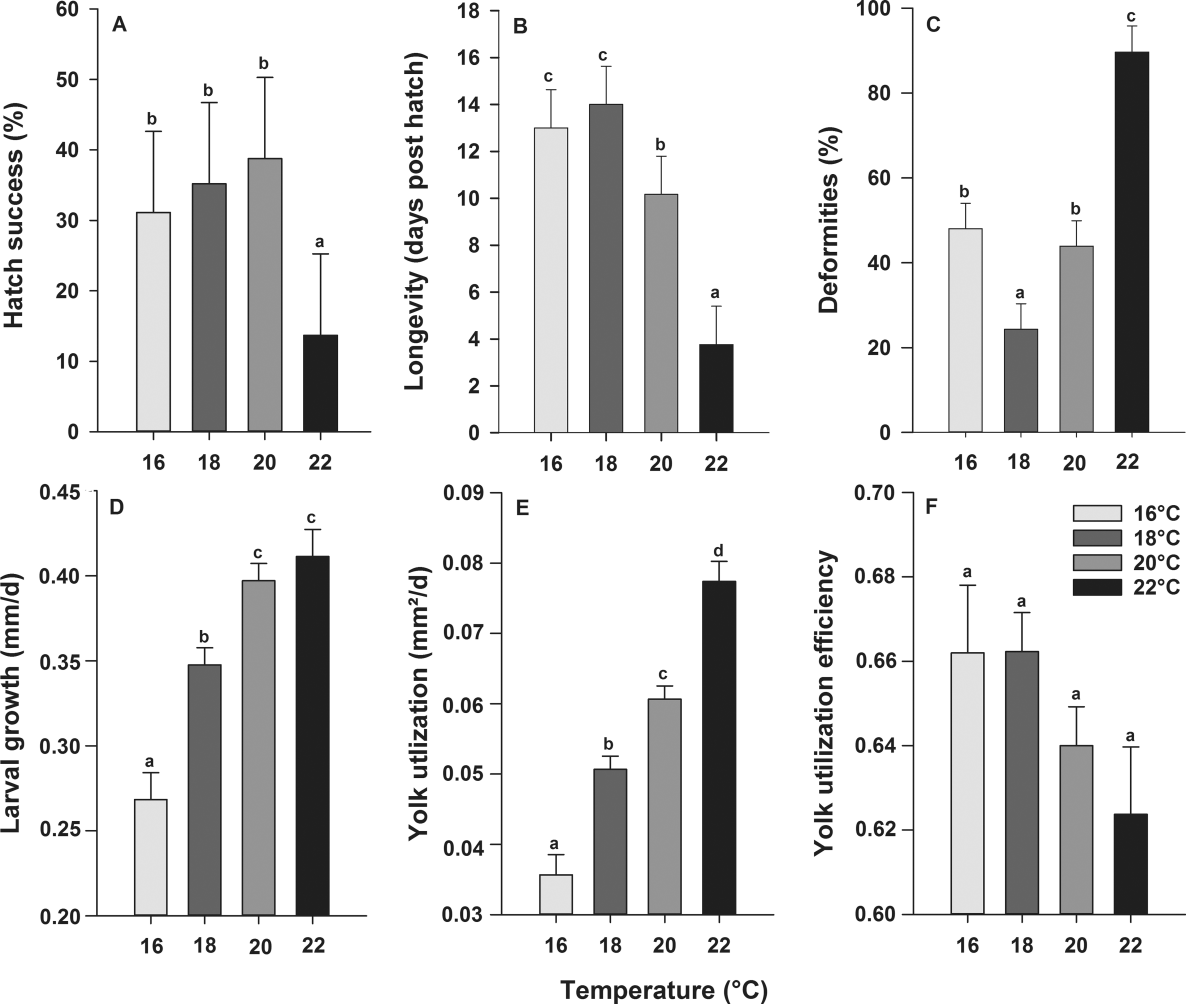
**Effect of rearing temperature on larval European eel (*Anguilla anguilla*)** (A) hatch success, (B) survival, (C) deformities at hatch, (D) growth rate, (E) yolk utilization and (F) yolk utilization efficiency. Values represent means (± SEM) among four crosses at each temperature. Means were contrasted using the Tukey-Kramer method and treatments with the same letters are not significantly different (P > 0.05).

Generally, development was delayed when embryos and larvae were reared in cold temperatures while accelerated in warm temperatures (Fig 1). Developmental rates were similar across all parental crosses investigated and larvae reached the first-feeding stages within 8 days or 232 hours post-fertilization (hpf) at 22°C compared to 10 days (288 hpf) at 20°C, 12 days (344 hpf) at 18°C or 16 days (456 hpf) at 16°C. Larval hatch success (Fig 2A) did not differ between 16, 18 and 20°C but significantly decreased at 22°C (P < 0.0001), while only two (in total) larvae hatched at 24°C, but they died shortly after hatch (100% mortality). Larval survival, in terms of longevity (Fig 2B), was lowest at 22°C (4 ± 2 dph) and highest at 18°C (14 ± 2 dph). Temperature had a significant influence on the incidence of larval deformities at hatch (P < 0.0001), where larvae reared at 18°C showed significantly less deformities (24 ± 6%), compared to 16°C (48 ± 6%), 20°C (44 ± 6%) or 22°C (90 ± 6%) (Fig 2C).

### Larval growth, yolk utilization and yolk utilization efficiency

Temperature had a significant influence (P < 0.0001) on larval growth rate. Here, larval growth increased with increasing temperature from 0.27 ± 0.02 at 16°C to 0.41 ± 0.02 mm d^-1^ at 22°C (Fig 2D). Similarly, temperature had a significant impact (P < 0.0001) on YU, which increased with increasing temperature, from 0.04 ± 0.003 mm^2^ d^-1^ at 16°C to 0.08 ± 0.003 mm^2^ d^-1^ at 22°C (Fig 2E). Temperature did not significantly influence YUE which decreased with increasing temperature, from 0.66 ± 0.02 mm^2^ d^-1^ at 16°C and 18°C to 0.62 ± 0.02 mm^2^ d^-1^ at 22°C (Fig 2F).

### Larval length

**Fig 3:**
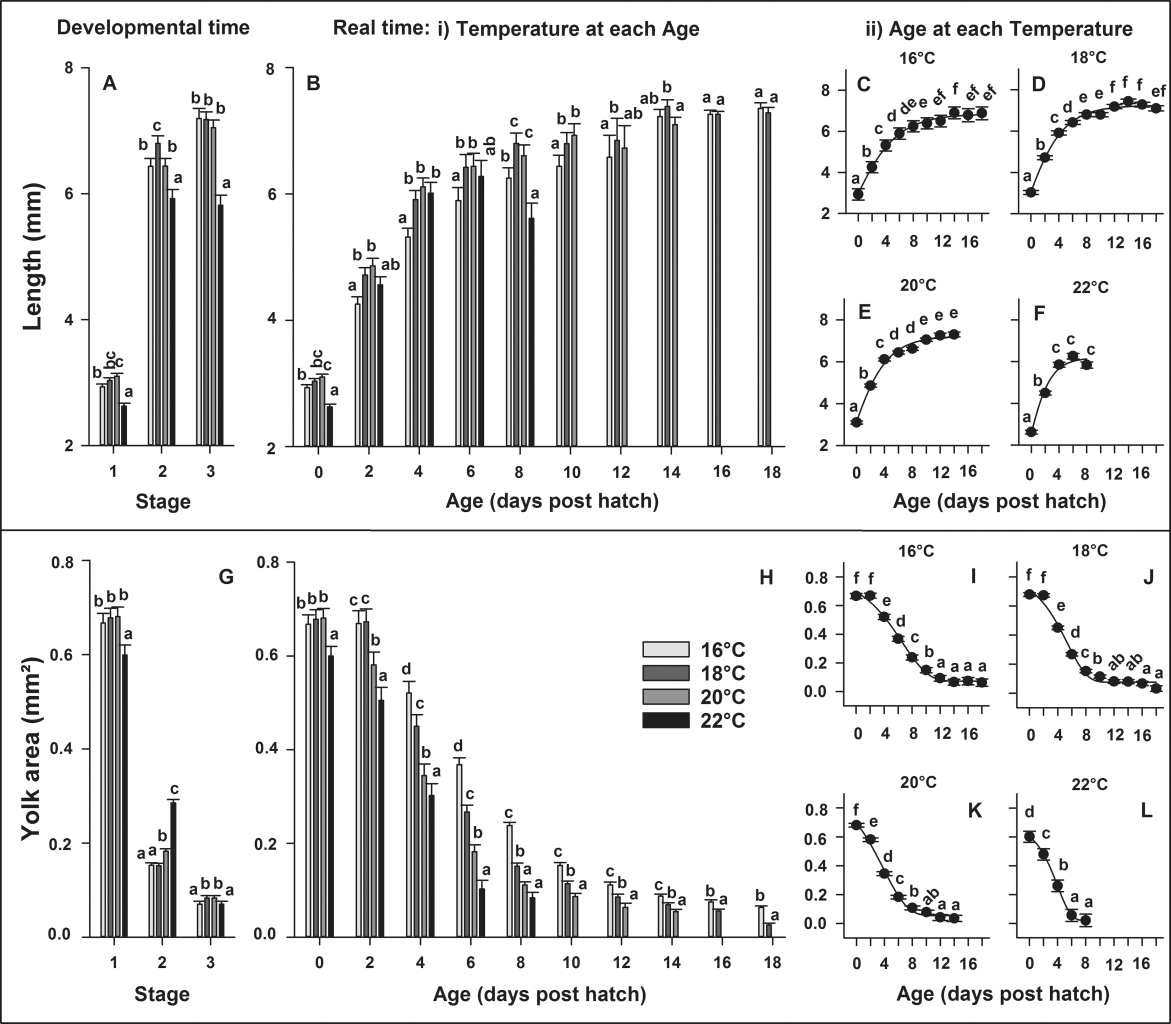
**Effect of rearing temperature on larval European eel (*Anguilla anguilla*)** length (A) or yolk area (G) at specific developmental Stages (1, 2 and 3) and length (B) or yolk area (H) in real time, as well as effect of age on length (C-F) or yolk area (I-L) at each temperature. Exponential regressions explain the relationship between age and length (C-F; P < 0.025, R^2^ > 0.98) as well as between age and yolk area (I-L; P < 0.006, R^2^ = 0.99) at all temperatures. Values represent means (± SEM) among four crosses at each temperature. Treatments with the same letters are not significantly different (P > 0.05).

Significant differences in length between temperatures occurred at all developmental stages investigated (P < 0.0001), where larvae reared at 22°C were smaller compared to all the other temperature treatments (Fig 3A). Larvae reared at 16, 18 and 20°C did not differ in length in Stage 3 (first-feeding), though significant differences occurred at the earlier developmental Stages (1 and 2). In real time, we observed a temperature × age interaction (P < 0.0001), thus the model was decomposed into a series of reduced ANOVA models to determine the effect of temperature for each age (Fig 3B) and of age for each temperature (Fig 3C–3F). Significant differences in length among temperatures occurred throughout development on 0, 2, 4, 6, 8, 10, 12 and 14 dph (P < 0.003). Typically, larvae reared in the colder (16°C) and warmer (22°C) thermal treatments were significantly smaller than larvae reared in the intermediate temperatures of 18 and 20°C. Larvae reared at 16°C grew to sizes similar to larvae reared at 18°C or 20°C (14–18 dph). Within each temperature, larval age significantly influenced length when larvae were reared at 16, 18, 20 and 22°C (P < 0.0001). Relationships between age and larval length can be explained by exponential regressions (B-E) at 16°C [y = 6.85 * exp(-exp(-(x + 0.63) / 3.54)), R^2^ = 0.99], 18°C [y = 7.24 * exp(-exp(-(x + 0.45) / 2.94)), R^2^ = 0.99], 20°C [y = 7.23 * exp(-exp(-(x + 0.53) / 2.79)), R^2^ = 0.99] and 22°C [y = 6.19 * exp(-exp(-(x + 0.22) / 1.74)), R^2^ = 0.98].

### Larval yolk area

In developmental time (Fig 3G), significant differences in yolk area among temperatures occurred at all developmental stages investigated (P < 0.008). Larvae reared at 22°C had most yolk reserves on Stage 2 but least yolk reserves on Stage 1 (hatch) or Stage 3 (first-feeding) compared to the other temperature treatments. Larvae reared at 16, 18 and 20°C did not differ in yolk area on Stage 1, though significant differences occurred at the later developmental Stages (2 and 3) investigated. In real time, yolk area was significantly affected by the temperature × age interaction (P < 0.0001) so the model was again decomposed to determine the effect of temperature for each age (Fig 3H) and of age for each temperature (Fig 3I–3L). Significant differences in yolk area among temperatures occurred throughout development on 0, 2, 4, 6, 8, 10, 12, 14, 16 and 18 dph (P < 0.002). The warmer the rearing temperature, the faster the YU and less yolk reserves were left for larvae to utilize. Within each temperature, larval age significantly influenced yolk area when larvae were reared in 16, 18, 20 and 22°C (P < 0.0001). Relationships between age and yolk-sac area can be explained by exponential regressions (G-J) at 16°C [y = 0.07 + 0.74 * exp(-exp(-(x—6.60) / - 3.71)), R^2^ = 0.99], 18°C [y = 0.08 + 0.74 * exp(-exp(-(x– 5.36) / - 2.82)), R^2^ = 0.99], 20°C [y = 0.05 + 0.89 * exp(-exp(-(x—3.85) / - 3.43)), R^2^ = 0.99] and 22°C [y = 0.02 + 0.66 * exp(-exp(-(x—3.97) / - 1.94)), R^2^ = 0.99].

### Molecular analyses

Gene expression of selected genes was compared across temperature treatments in real time and at specific developmental stages (developmental time). In real time, the expression of target genes was affected by both larval age and temperature (see specific genes below). Even though the expression profiles appeared very similar in real time and only shifted with temperature, in developmental time, differences occurred across developmental stages and among temperatures (see specific genes below).

#### Heat shock proteins

**Fig 4:**
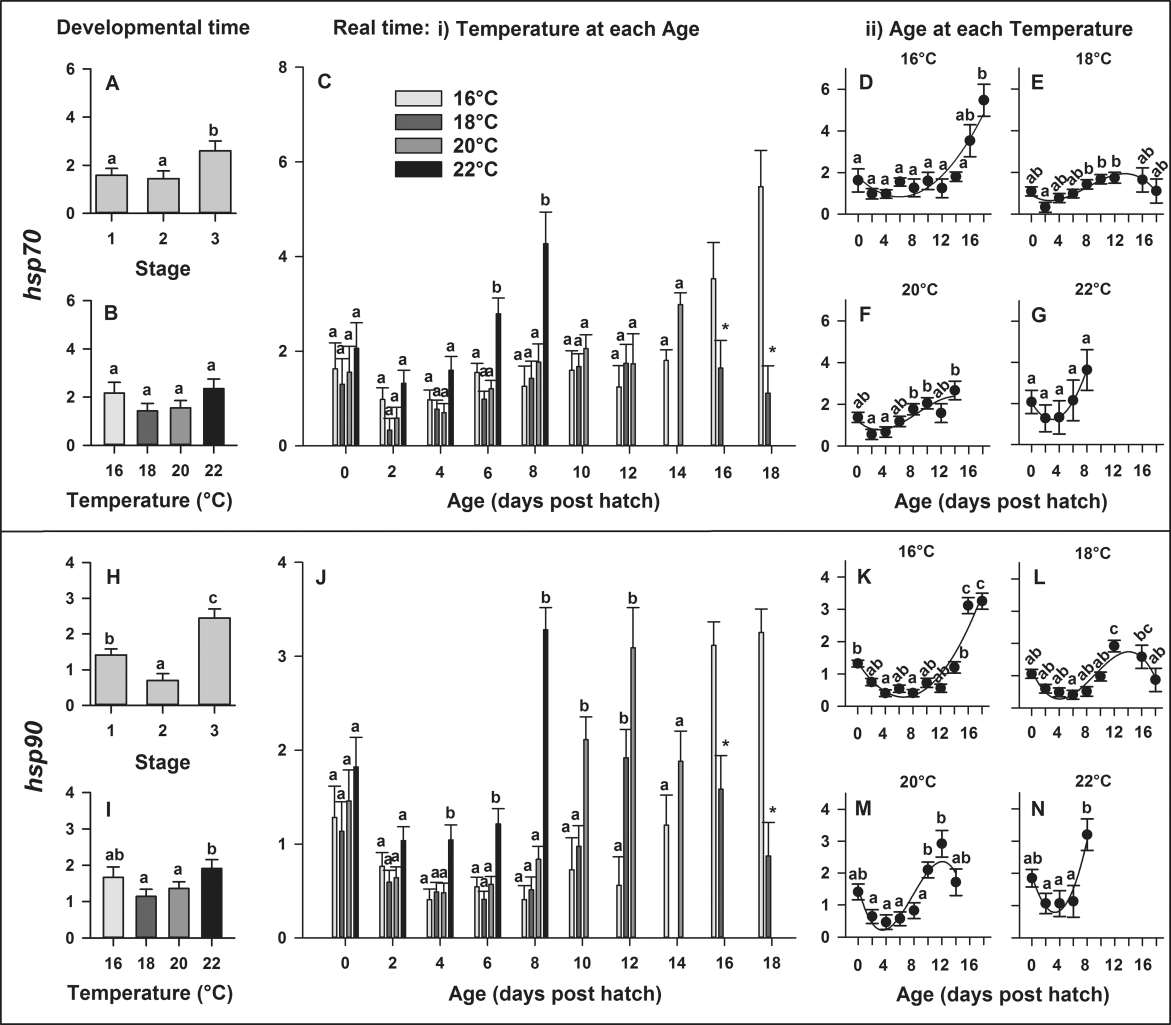
**Effect of rearing temperature on larval European eel (*Anguilla anguilla*) gene expression** of *hsp70* (A-B) or *hsp90* (H-I) at specific developmental Stages (1, 2 and 3) and *hsp70* (C) or *hsp90* (J) in real time, as well as effect of age on *hsp70* (D-G) or *hsp90* expression (K-N) at each temperature. Relationships between age and *hsp70* expression can be explained by quadratic regressions at 16 or 22°C and by cubic regressions at 18 or 20°C (P < 0.03, R^2^ > 0.78). Relationships between age and *hsp90* expression can be explained by quadratic regressions at 16 or 22°C and by cubic regressions at 18 or 20°C (P < 0.001, R^2^ > 0.85). Data points with an asterisk (*) were not included in the statistical model due to insufficient sample size. Values represent means (±SEM) among four crosses at each temperature and treatments with the same letters are not significantly different (P > 0.05).

In developmental time, expression of *hsp70* significantly (P = 0.015) increased on Stage 3 (first-feeding) but did not significantly differ across temperatures (P = 0.066; Fig 4A and 4B). In real time, gene expression of *hsp70* was significantly affected by the temperature × age interaction (P = 0.0001; Fig 4C). Therefore, the model was, as above, decomposed. Significant differences in gene expression of *hsp70* between temperatures occurred on 6 and 8 dph (P < 0.010). Expression levels of *hsp70* increased up to 5-fold at the colder (16°C) and 4-fold at the warmer (22°C) suboptimal thermal limit, while expression levels at 18 and 20°C remained lower than in the other temperatures throughout ontogenetic development. Larval age significantly influenced gene expression of *hsp70* when larvae were reared at 16, 18 and 20°C (P < 0.003; Fig 4D–4G) and the relationships between age and *hsp70* expression were best explained by quadratic parabola regressions (D, G) at 16°C (y = 1.81–0.33 x + 0.03 x^2^, R^2^ = 0.88) and 22°C (y = 2.06–0.56 x + 0.09 x^2^, R^2^ = 0.99) and by cubic sigmoidal regressions (E-F) at 18°C (y = 0.93–0.22 x + 0.05 x^2^ - 0.002 x^3^, R^2^ = 0.89) and 20°C (y = 1.21–0.30 x + 0.06 x^2^ - 0.002 x^3^, R^2^ = 0.78).

In developmental time, expression of *hsp90* significantly increased on Stage 1 (hatch), decreased on Stage 2 and increased again on Stage 3 (first-feeding) (P < 0.0001; Fig 4H). Moreover, *hsp90* expression was significantly lower at 18 and 20°C, elevated at colder and warmer temperatures and peaked at 22°C (P < 0.03; Fig 4I). In real time, gene expression of *hsp90* was significantly affected by the temperature × age interaction (P < 0.0001) and again the model was as previously decomposed. Significant differences in gene expression of *hsp90* among temperatures occurred throughout development on 4, 6, 8, 10 and 12 dph (P < 0.02; Fig 4J). Expression levels of *hsp90* increased up to 3-fold at 16, 20 and 22°C, while expression levels at 18°C remained (similarly to *hsp70*) lower than for the other temperatures throughout development. Larval age influenced gene expression of *hsp90* when larvae were reared in 16, 18, 20 and 22°C (P < 0.010; Fig 4K–4N) and relationships between developmental age and *hsp90* expression were best explained by quadratic parabola regressions at 16°C (y = 1.35–0.32 x + 0.02 x^2^, R^2^ = 0.91) and 22°C (y = 1.93–0.69 x + 0.10 x^2^, R^2^ = 0.93) and by cubic sigmoidal regressions at 18°C (y = 1.18–0.49 x + 0.08 x^2^ - 0.003 x^3^, R^2^ = 0.85) and 20°C (y = 1.57–0.86 x + 0.16 x^2^ - 0.01 x^3^, R^2^ = 0.85).

#### Growth hormone and insulin-like growth factors

**Fig 5:**
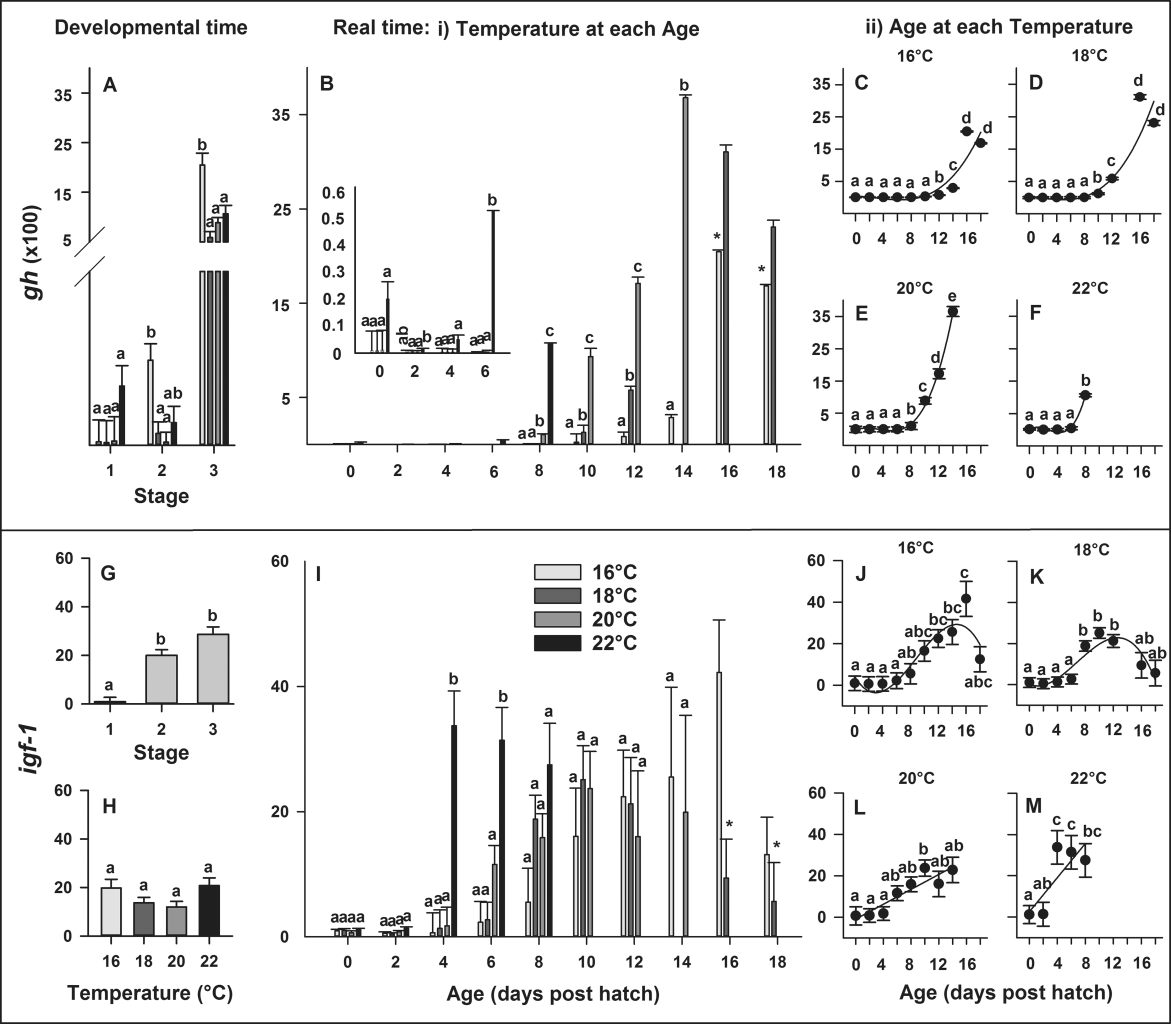
**Effect of rearing temperature on larval European eel (*Anguilla anguilla*) gene expression** of *gh* (A) or *igf-1* (G-H) at specific developmental Stages (1, 2 and 3) and *gh* (B) or *igf-1* (I) in real time, as well as effect of age on *gh* (C-F) or *igf-1* expression (J-M) at each temperature. Relationships between age and *gh* expression can be explained by cubic sigmoidal regressions at all temperatures (P < 0.0001, R^2^ > 0.82). The relationship between age and *igf-1* expression can be explained by cubic sigmoidal regressions at 16 or 18°C and by linear regressions at 20 or 22°C (P < 0.002, R^2^ > 0.64). Data points with an asterisk (*) were not included in the statistical model due to insufficient sample size. Values represent means (± SEM) among four crosses at each temperature and treatments with the same letters are not significantly different (P > 0.05).

In developmental time, significant differences among temperatures occurred at Stage 2 and 3, where larvae reared at the colder thermal limit (16°C), expressed higher levels of *gh* compared to the warmer temperatures (P < 0.006; Fig 5A). In real time, gene expression of *gh* was significantly affected by the temperature × age interaction (P < 0.0001). Thus, the model was decomposed. Significant differences in gene expression of *gh* among temperatures occurred throughout development on 2, 6, 8, 10, 12 and 14 dph (P < 0.03; Fig 5B). Expression levels of *gh* increased up to 1000-fold at 22°C, 2000-fold at 16°C and more than 3000-fold at 18 and 20°C. Larval age significantly influenced gene expression of *gh* at all temperatures (P < 0.0001; Fig 5C–5F). Here, relationships between developmental age and *gh* expression were best explained by cubic sigmoidal regressions at 16°C (y = 36.21–5.24 x—6.49 x^2^ + 0.72 x^3^, R^2^ = 0.82), 18°C (y = 40.99–23.52 x—3.18 x^2^ + 0.75 x^3^, R^2^ = 0.86), 20°C (y = - 14.29 + 79.62 x—33.28 x^2^ + 3.29 x^3^, R^2^ = 0.99) and 22°C (y = 4.02 + 147.59 x—81.17 x^2^ + 9.89 x^3^, R^2^ = 0.99).

In developmental time, expression of *igf-1* significantly increased with developmental stage (P < 0.0001; Fig 5G) but did not significantly differ across temperatures (P = 0.081; Fig 5H). In real time, gene expression of *igf-1* was affected by the temperature × age interaction (P = 0.001), thus the model was again decomposed. Differences in gene expression of *igf-1* among temperatures occurred during early development on 4 and 6 dph (P < 0.001; Fig 5I). Expression levels of *igf-1* increased up to 40-fold at 16°C and 30-fold at 22°C, indicating the colder and warmer suboptimal thermal limits respectively. Larval age significantly influenced gene expression of *igf-1* when larvae were reared in 16, 18, 20 and 22°C (P < 0.002; Fig 5J–5M). Here, the relationships between developmental age and *igf-1* expression were best explained by cubic regressions at 16°C (y = 4.18–5.53 x + 1.11 x^2^ - 0.04 x^3^, R^2^ = 0.82) or 18°C (y = 0.19–1.39 x + 0.65 x^2^ - 0.03 x^3^, R^2^ = 0.82) and by linear regressions at 20°C (y = - 0.92 + 1.79 x, R^2^ = 0.84) or 22°C (y = 2.45 + 4.14 x, R^2^ = 0.64).

**Fig 6:**
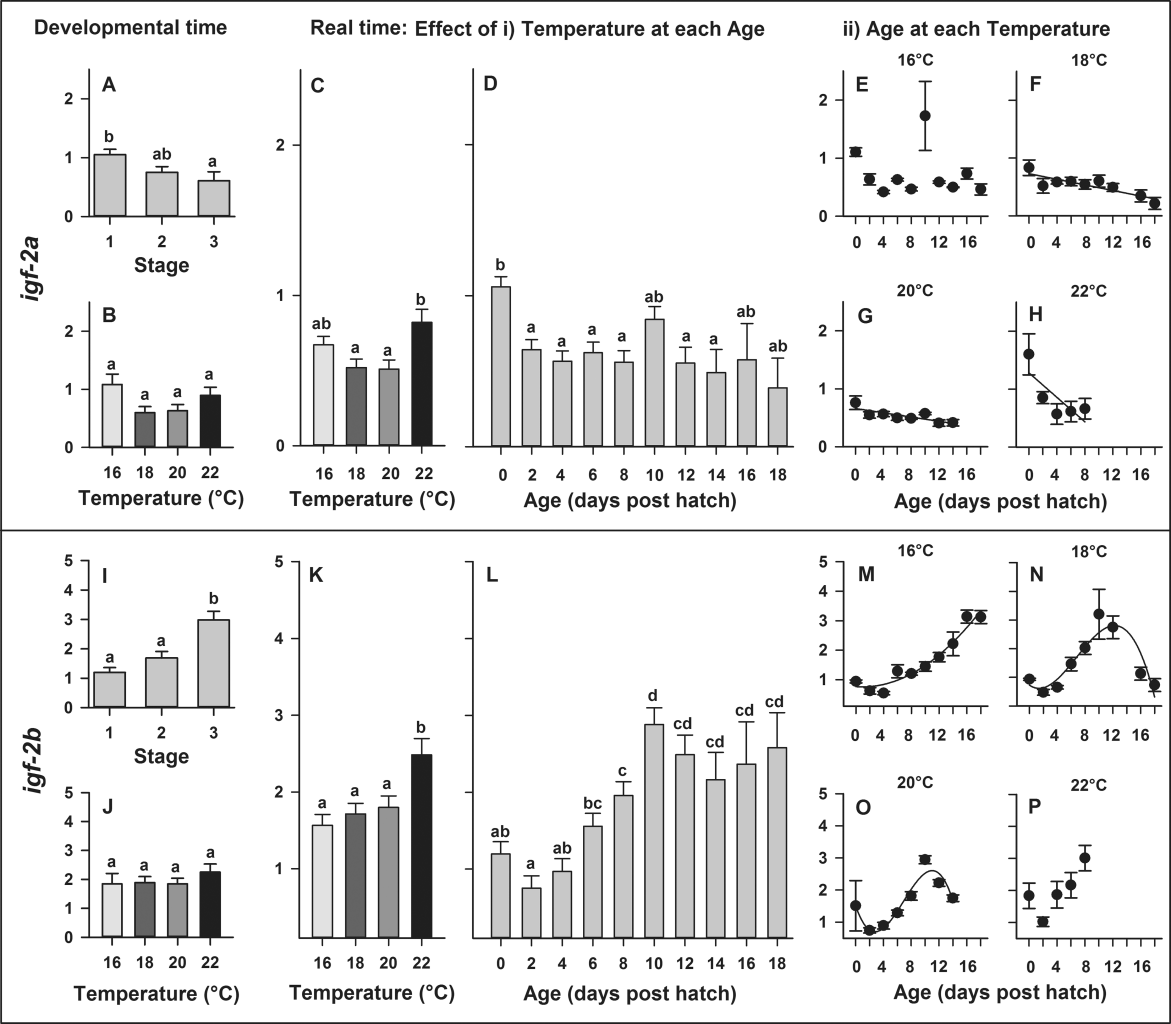
**Effect of rearing temperature on larval European eel (*Anguilla anguilla*) expression** of *igf-2a* (A-B) or *igf-2b* (I-J) at specific developmental Stages (1, 2 and 3) and i*gf-2a* (C-D) or *igf-2b* (K-L) in real time, as well as effect of developmental age on *igf-2a* (C-F) or on *igf-2b* (I-L) expression at each temperature. Relationships between developmental age and *igf-2a* expression can be explained by linear regressions at 18, 20 and 22°C (P < 0.025, R^2^ > 0.61; Fig F-H). Relationships between developmental age and *igf-2b* expression can be explained by a quadratic parabola regression (M) at 16°C and by cubic sigmoidal regressions (N-O) at 18°C and 20°C (P < 0.0001, R^2^ > 0.84; Fig 6I–6K). Values represent means (± SEM) among four crosses at each temperature and treatments with the same letters are not significantly different (P > 0.05).

In developmental time, expression of *igf-2a* significantly decreased with developmental stage (P = 0.020; Fig 6A) but did not significantly differ across temperatures (P = 0.061; Fig 6B). In real time, gene expression of *igf-2a* was significantly affected by temperature (P = 0.007) and larval age (P < 0.0001), though no significant temperature × age interaction (P = 0.1288) was detected. Gene expression of *igf-2a* significantly increased at 22°C (Fig 6C) and significantly decreased throughout ontogeny with increasing larval age (Fig 6D). The relationships between developmental age and *igf-2*a expression were best explained by linear regressions (Fig 6F–6H) at 18°C (y = 0.73–0.02 x, R^2^ = 0.74), 20°C (y = 0.66–0.02 x, R^2^ = 0.65) and 22°C (y = 1.28–0.11 x, R^2^ = 0.61), while no significant relationship between developmental age and *igf-2a* expression was detected at 16°C (Fig 6E).

In developmental time, expression of *igf-2b* significantly increased with developmental stage (P < 0.0001; Fig 6A) but did not significantly differ across temperatures (P = 0.658; Fig 6I–6J). In real time, gene expression of *igf-2*b was significantly affected by temperature (P < 0.001) and larval age (P < 0.0001), while no interaction was detected between temperature and age (P = 0.112). Gene expression of *igf-2b* significantly increased at 22°C (Fig 6K) and significantly increased throughout ontogeny with increasing larval age (Fig 6L). The relationships between developmental age and *igf-2b* expression were best explained by a quadratic regression at 16°C (y = 0.76–0.02 x + 0.01 x^2^, R^2^ = 0.95) and by cubic regressions at 18°C (y = 0.73–0.17 x + 0.07 x^2^ - 0.003 x^3^, R^2^ = 0.84) or 20°C (y = 1.51–0.63 x + 0.14 x^2^ - 0.01 x^3^, R^2^ = 0.91), while no significant relationship between developmental age and *igf-22* expression was detected at 22°C (Fig 6M–6P).

## Discussion

This study identified the thermal tolerance range and limits of European eel early life history and elucidated thermally induced phenotypical changes as well as changes in the interlinked expression of genes associated to early development in fish. Temperature influenced all traits investigated. Larvae generally developed and grew throughout ontogeny until the first-feeding stages by utilizing their yolk reserves in all temperature treatments, except at 24°C which was found to be the deleterious upper thermal limit. Generally, increasing temperature caused acceleration in development and the higher the temperature, the earlier the expression response of any specific targeted gene. In more detail, larval yolk utilization and growth rates increased, while yolk utilization efficiency decreased with increasing temperature. Furthermore, temperature influenced hatch success, time to hatch, deformities at hatch, larval survival and expression of targeted genes relating to larval development (*gh* and *igf*) and stress (*hsp*). Here, the expression of targeted genes was affected by larval age/stage and temperature, as well as their interaction when compared in both, real or relative time.

### Larval morphology

We observed a reduced larval stage duration with increasing temperature, where larvae reached the feeding stages within 8 days at 22°C compared to 16 days at 16°C; resulting in 50% faster development. A similar reduced stage duration with increasing temperature during ELH, has been previously shown in other important fish species such as haddock (*Melanogrammus aeglefinus*) [48], brown trout (*Salmo trutta*) [49], Atlantic and Baltic cod, *Gadus morhua* [50–52], Northern rock sole, *Lepidopsetta polyxystra* [53] and Atlantic herring, *Clupea harengus* [54]. Generally and within the thermal tolerance window, larval stage duration is reduced at higher temperatures due to faster growth [53, 54]. However, unfavorable thermal conditions close to the thermal tolerance limits are known to cause less efficient yolk utilization and reduced growth [25]. This phenomenon was also observed in this study, further validating the fact that increased temperature, especially towards unfavorable limits, results in less efficient conversion of yolk into somatic tissue. Moreover, we observed the lowest yolk utilization efficiency, combined with the most deformities at hatch when reared at 22°C, pointing out an upper thermal plasticity limit. Limited thermal plasticity has been correlated with the inability to effectively make physiological adjustments to achieve homeostasis under elevated temperatures, resulting in chronic thermal stress of delta smelt, *Hypomesus transpacificus* [55]. Moreover, no larvae survived at 24°C, representing a deleterious upper thermal limit, while larval deformities at hatch increased at 16°C, representing a colder thermal tolerance limit during early development of European eel.

The rearing temperature (20–21°C) used in most current protocols for hatchery production of *A*. *anguilla* larvae [3, 40], is similar to what is being used to produce hybrid larvae from male *A*. *anguilla* and female *A*. *australis* [21]. Hybrid larvae between male *A*. *anguilla* and female *A*. *japonica* have also been experimentally produced and reared at 21–22°C [19], while the closely related *A*. *rostrata* larvae have been reared at 20°C after assisted reproduction [17]. Moreover, an attempt to rear *A*. *australis* or *A*. *australis* × *A*. *dieffenbachii* hybrid larvae was undertaken at a thermal regime of 18.2–22.7°C [20]. The thermal regimes for all the above mentioned eel species and their hybrid combinations seem to be rather concurrent with the thermal tolerance range identified in this study (16–22°C). Though, we here revealed a thermal optimum of 18°C, corresponding to the lowest incidence of larval deformities at hatch, highest larval survival and highest yolk utilization efficiency. This finding provides important information towards improving the conditions for larval rearing and production success of this species in aquaculture. Furthermore, the only eel species with a successfully closed life cycle in captivity is *A*. *japonica*, where hatchery produced larvae are reared at 25°C [15]. This thermal optimum is far above the temperatures used for all the other eel species mentioned above, including this study, though it seems to be close to the thermal regime of the recently identified natural spawning area of this species [16].

### Gene expression

In the present study, we observed an increased expression of *hsp70* and *hsp90* with increasing age and stage, while expression levels increased towards both, colder (16°C) and warmer (22°C) thermal limits. The expression of *hsp* has previously been linked to phenotypic variation (deformities) of green sturgeon (*Acipenser medirostris*) larvae in response to thermal stress, pointing out the importance of this cellular mechanism associating HSPs with the organism’s thermal vulnerability [32]. Similarly, individual gene expression of specific molecular chaperones such as *hsp70* and *hsp47* was up-regulated in response to thermal stress, while others such as *hsp90* remained at constitutive levels across all treatments in larval delta smelt [55]. Therefore, our findings support the fact that HSP’s are linked to phenotypic variation in the response and vulnerability of larvae to thermal stress. Furthermore, HSP function and response to stress is now recognized to be universal to all cells and not restricted to heat stress [30]. Nutritional status, for instance, can further affect HSP responses to thermal stress and it has previously been shown that *hsp* expression can be related to feed deprivation in larval fish [56]. Generally, expression levels of both *hsp’s* investigated in this study peaked around the first-feeding stages, potentially representing a combined effect of thermal stress and developmental preparation towards exogenous feeding. Thus, it would be interesting to further investigate this phenomenon in the future, by comparing phenotypic and molecular differences between exogenously first-feeding and starving larvae. Moreover, it would be of interest to investigate expression levels of *hsp’s* when larvae are reared under different conditions and not in experimental flasks or chambers. Nevertheless though, expression levels of both genes at 18–20°C remained lower compared to expression at the other temperatures during larval development, which in combination with occurrence of the lowest number of deformities most probably represents a more optimal thermal environment for rearing European eel offspring.

The expression of *gh*, one of the somatotropic axis actor genes, was in this study shown to be influenced by temperature during larval (pre-leptocephalus) development of European eel. It has previously been observed in fish, that GHs are involved in most physiological processes such as metabolism, and growth [31] and that they can be temperature sensitive [57]. In our study, *gh* was up-regulated and expression peaked at 18–20°C, probably representing the most optimal environment for larval growth. Moreover, we observed an increase in *gh* expression with larval developmental age, though we did not determine the location of *gh* regulation. Expression of *gh* has been shown to be regulated in the liver of juveniles and adults, or in the brain of larval stages of gilthead sea bream, *Sparus aurata* [58]. Similarly, expression of *gh* was relatively weak during milkfish (*Chanos chanos*) embryogenesis and hatching but increased during larval development starting on day 2 post hatch, implying an endogenous production by the larval pituitary gland and coinciding with the period of accelerated larval growth [59]. The timing of ontogeny and functionality of the European eel pituitary gland has not been completely documented yet, but an immunohistochemical study in this species suggests that the majority of pituitary cells differentiate before metamorphosis to the glass eel stage, presumably during the leptocephalus stages [60]. However, the first immunohistochemical GH signal in the ricefield eel, *Monopterus albus*, has been recently detected as early as 3 dph [61]. Most likely, the sharp increase of *gh* expression observed in our study corresponds to the timing of pituitary GH secreting cells functionality. Although it needs to be further documented by immunohistological data, our results would indicate the presence of GH secreting cells during European eel early life ontogeny, much earlier than previously anticipated; an increased expression was already observed at 4–6 dph. That would also be in accordance with the results of a study of the closely related Japanese eel, where the *gh* transcripts and the production of GH protein were detected at 6 dph (when reared at 23°C), suggesting an important role of GH in larval growth and survival before the leptocephalus stage [62].

IGF*s*, the other group of acting genes in the somatotropic axis and thus closely connected and regulated by GH, are known to be stimulated by temperature and associated among others with growth, metabolism and development [31]. In our study, we observed an increased expression of *igf-1* and *igf-2b* with increasing larval age/stage at all temperatures investigated, signifying the involvement of the somatotropic axis genes during larval development of European eel. Furthermore, transcripts for *igf’s* have been detected throughout development in unfertilized eggs, embryos, and larvae of several other fish species such as Senegalese sole (*Solea senegalensis*), zebrafish (*Danio rerio*), sea bass (*Dicentrarchus labrax*), gilthead seabream (*Sparus aurata*) and rabbitfish (*Siganus guttatus*), suggesting that they can be products of maternal as well as embryonic genomes and that *igf-1* and *igf-2* may thus regulate early development of teleosts [63–66]. Similarly, in a recent study correlating gene expression to healthy embryonic development and hatch success in European eel, it was shown that expression levels of *igf-2* increased during embryogenesis until larval hatching [67]. Interestingly, in our study, we observed an increased expression of *igf-2a* and *igf-2b* at hatch, strengthening the theory of the involvement of *igf’s* during the hatching process of European eel.

### Perspective

From an aquaculture perspective, this study determined the thermal tolerance limits and identified a more optimal intermediate thermal environment (18–20°C), with efficient growth and fewer deformities, for future rearing of ELH stages of European eel. The next step would be to further optimize other environmental rearing conditions, such as salinity regime (similar to that used in Japanese eel culture) and combine this information with our existing knowledge (light and temperature). Together, these enhanced rearing conditions will provide a promising step towards the sustainable culture of this species. So far, the intermediate thermal optimum, identified here, is colder than the optimal thermal rearing conditions (25°C) of the closely related Japanese eel [15]. Currently, the Japanese eel is the only eel species with a closed life cycle in captivity and the experimentally identified optimum thermal conditions are found to be similar to those encountered in their recently identified natural spawning area [15, 16]. In this regard, a recent study has shown that inducing vitellogenesis at 15°C and final maturation at 18°C, results in higher reproductive performance of European eel [68]. These results indicate that European eel reproductive maturation might occur in the deeper and colder layer while gamete development in a slightly warmer layer of the Sargasso Sea. Thus, the thermal tolerance limits identified in this study might not only advance the rearing conditions for future culture of European eel larvae but possibly also contribute to the understanding for further hypotheses regarding the natural spawning conditions and location. As such, the results of this study suggest that the habitat (or niche) of the earliest life stages of European eel in nature might be the characteristic “18°C” ocean layer of the Sargasso Sea [7]. However, extrapolation of lab work to the field should always be approached carefully. Thus, the mystery of the exact location of European eel early life stages and their preferred conditions in nature still remains an enigma. However, we clearly show that increasing temperature had a deleterious impact on European eel embryonic and larval development and survival. Thus, our results support the recent hypotheses, that rising temperatures in the Sargasso Sea (linked to factors such as North Atlantic Oscillation and food availability) might show negative effects on eel recruitment [69–71].

### Conclusion

In conclusion, this study provides important insights on phenotypic sensitivity to temperature and the underlying gene expression of the associated molecular mechanisms in European eel larvae. Temperature was found to influence all traits investigated, resulting in reduced larval stage duration at higher temperatures due to accelerated development, but with decreasing yolk utilization efficiency. The highest larval survival, combined with the lowest incidence of larval deformities at hatch and the highest yolk utilization efficiency that occurred when reared at 18°C, as well as the correspondence with a high *gh* and low *hsp* expression, indicate a more optimal environment for early life development and rearing. Understanding the biological responses, limits and adaptabilities or preferences to extrinsic environmental factors, such as temperature, provides enhanced knowledge for the optimization of rearing techniques of a socially and economically important species such as European eel, as well as insights into its ecology.
